# The Impact of High-Dose Fish Oil Supplementation on Mfsd2a, Aqp4, and Amyloid-β Expression in Retinal Blood Vessels of 5xFAD Alzheimer’s Mouse Model

**DOI:** 10.3390/ijms25179400

**Published:** 2024-08-29

**Authors:** Irena Jovanovic Macura, Desanka Milanovic, Vesna Tesic, Tamara Major, Milka Perovic, Miroslav Adzic, Sanja Ivkovic

**Affiliations:** 1 Institute for Biological Research “Sinisa Stankovic”, National Institute of Republic of Serbia, University of Belgrade, 11000 Belgrade, Serbia; irena.macura@ibiss.bg.ac.rs (I.J.M.); desan@ibiss.bg.ac.rs (D.M.); vesna.tesic@lsuhs.edu (V.T.); milkap@ibiss.bg.ac.rs (M.P.); 2 Faculty of Pharmacy, University of Belgrade, 11000 Belgrade, Serbia; majtamara@gmail.com; 3 Vinca–Institute for Nuclear Sciences, National Institute of Republic of Serbia, University of Belgrade, 11000 Belgrade, Serbia; miraz@vin.bg.ac.rs

**Keywords:** Mfsd2a, Aqp4, retina, Alzheimer’s disease, BRB, transcytosis, amyloid beta

## Abstract

In patients with Alzheimer’s disease (AD) and in animal models, the increased accumulation of amyloid β (Aβ) in retinal blood vessels strongly correlates with brain amyloid deposits and cognitive decline. The accumulation of Aβ in blood vessels may result from impaired transcytosis and a dysfunctional ocular glymphatic system in AD. High-dose fish oil (FO) supplementation has been shown to significantly change the expression of major facilitator superfamily domain-containing protein 2a (Mfsd2a), a key regulator of transcytosis, and Aquaporin 4 (Aqp4), an essential component of the glymphatic system in the retinas of WT mice. We examined the expression of Mfsd2a and Aqp4 in the retinas of 4-month-old 5xFAD female mice supplemented with high-dose FO for three weeks. There was a significant increase in Mfsd2a expression in 5xFAD retinas supplemented with FO compared to control 5xFAD mice. Additionally, the increase in Aqp4 expression observed in 4-month-old 5xFAD retinas, indicative of an impaired glymphatic system, was significantly decreased. Simultaneously, Aβ accumulation in 5xFAD retinal blood vessels was reduced following FO supplementation. These findings suggest that high-dose FO supplementation could serve as an adjunct in developing new treatments aimed at improving the regulation of transcytosis or the function of the glymphatic system in the AD retina.

## 1. Introduction

Vascular dysfunction in the brain is acknowledged as a crucial contributor to cognitive decline in Alzheimer’s disease (AD) [1,2]. Key features of AD pathology include cerebral amyloid angiopathy (CAA) [3] and disruption of the blood–brain barrier (BBB) [4,5]. CAA, present in more than 85% of AD cases, is characterized by the pathological deposition of amyloid β (Aβ) on blood vessels along with other vascular abnormalities, suggesting its potential as a clinical marker for AD [6]. Similar pathological changes occur in the retinas of individuals with Alzheimer’s disease (AD), where impaired blood–retinal barrier (BRB) function has been linked to increased vascular amyloidosis [7,8,9]. Notably, vascular pathologies in both cerebral and retinal tissues are strongly linked to cognitive deficits in AD animal models and patients [10,11]. In addition, vision abnormalities may appear earlier than the cognitive impairments [12].

The accumulation of amyloid in blood vessels is attributed to inadequate clearance of Aβ and is linked to impaired transcytosis and dysfunction in the glymphatic system. It was suggested that transcytosis, involving vesicular trafficking between the luminal and abluminal endothelial membranes, could be the major mechanism for BBB functioning [13,14]. The major facilitator superfamily domain-containing protein 2a (Mfsd2a) was shown to be critical in the regulation of transcytosis [15,16], and decreased levels of Mfsd2a expression on retinal blood vessels were associated with aging and AD pathology [17]. Another mechanism involved in the accumulation of Aβ is the dysfunctional glymphatic system [18,19]. An ocular lymphatic system, that depends on Aqp4-signaling for its proper function, was discovered in rodent models [20]. Aqp4, a transmembrane channel protein regulates the transport of fluids and metabolites between cells [21]. Besides the fact that the observed elevated expression levels of Aqp4 are associated with aging [22], these changes are also in correlation with the severity of CAA in the brain [23,24].

An especially high concentration of docosahexaenoic acid (DHA), the omega-3 long-chain polyunsaturated fatty acid (LC-PUFA), is a shared feature of both retina and brain. Besides DHA, proper retinal development and functions depends on other LC-PUFAs (both omega-3 and omega-6) [25,26]. The limited capacity of the retina to synthesize its own DHA demands that the retina obtains LC-PUFAs from other sources, mainly through food or different kinds of supplementation [27]. Another Mfsd2a function, besides regulating transcytosis, is to transport lipids at the surface of endothelial cells of the CNS, facilitating the delivery of DHA into the brain [28,29]. Consequently, the level of transcytosis and barrier penetrability is greatly regulated via the lipid composition of CNS endothelial cells plays [13]. In addition, the positive effects of n-3 PUFAs supplementation on the extracellular Aβ clearance are linked to the AQP4-dependent glymphatic system function [30,31]. 

Nevertheless, the observed lack of consistent therapeutic effects of the currently suggested supplementation doses was attributed to the inadequate final bioavailability [32]. Thus, supplementation with much higher doses of fish oil (FO) as a rich source of n-3 LC-PUFAs [33], even up to 10 g per day, has been increasingly recommended [32]. Additionally, high-dose FO supplementation during three weeks of treatment have significantly altered the expression levels of Mfsd2a in WT retinal blood vessels [34]. Importantly, studies showed that even a very short-term FO supplementation (1 and 3 week long) can have a strong effect on the pathology of Alzheimer’s disease (AD) in a mouse model [35,36,37]. 

In this study, we tested the effects of high-dose FO supplementation, lasting for three weeks, on the specific molecular changes and vascular amyloidosis in the retina of 5xFAD AD mouse model. The 5XFAD mice pathology include a significant increase in Aβ42/Aβ40 in the retina accompanied with vascular changes [38,39,40]. These changes, observed in three-month-old female 5xFAD mice, were suggested to correlate with similar findings in human AD [41]. Importantly, it was shown that retinal DHA is significantly lower in 5xFAD mice, in comparison to WT [42]. In this study, we assessed the outcomes of the short-term high-dose FO supplementation in the four-month-old female 5xFAD mice on the Mfsd2a, Aqp4, and cholesterol metabolism-related gene expression and on the Aβ retinal blood vessel accumulation. This study was conducted only on female mice, as previous reports revealed that female 5xFAD mice show stronger responses to treatments and genetic and environmental modifications [43].

## 2. Results

### 2.1. The Expression Levels of DHA Transporter Mfsd2a in 5xFAD Retinas Were Increased Following the High-Dose FO Treatment

**Figure 1:**
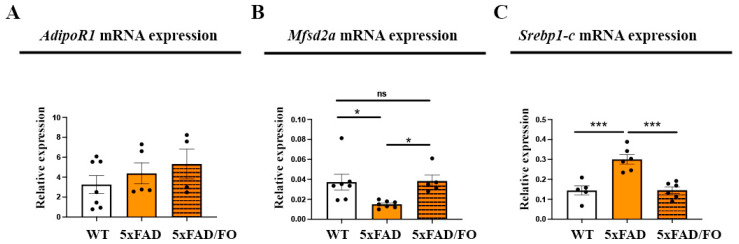
The expression levels of DHA transporter Mfsd2a in 5xFAD retinas were increased following the high-dose FO treatment. AdipoR1 (**A**), Mfsd2a (**B**), and Srebp1-c (**C**) mRNA expression in the control, 5xFAD, and 5xFAD-FO retinas. Data were analyzed using one-way ANOVA (with Tukey’s post hoc). The results are presented as mean ± SEM value. * *p* < 0.05, *** *p* < 0.001. ns = non-significant.

AdipoR1 and Mfsd2a have been recognized as primary mediators of DHA transport in the retina [44,45]. We aimed to determine whether the expression levels of these DHA transporters were affected by the high-dose FO supplementation (Figure 1A,B). The levels of AdipoR1 mRNA expression in 5xFAD retinas remained unaltered in comparison to the controls (Figure 1A), and FO supplementation did not affect its expression levels. The analyses of the expression levels of *Mfsd2a* showed that they are significantly lowered in 5xFAD retinas (2.46-fold decrease) and that the FO supplementation reversed this decrease and restored *Mfsd2a* expression to the control levels (Figure 1B).

Sterol regulatory element-binding protein 1-c (*Srebp1-c*), which participates in the regulation of the synthesis of fatty acids, is also suggested as a target of *Mfsd2a* expression [46,47]. The levels of *Srebp1-c* expression were elevated in the 5xFAD retinas (2.14-fold increase) in comparison to WT mice. However, they were significantly reduced with the FO treatment and reversed to the control levels (Figure 1C).

### 2.2. High-Dose FO Supplementation Did Not Alter the Expression of the Genes Involved in Regulating Cholesterol Synthesis and Transport in 5xFAD Retinas

**Figure 2:**
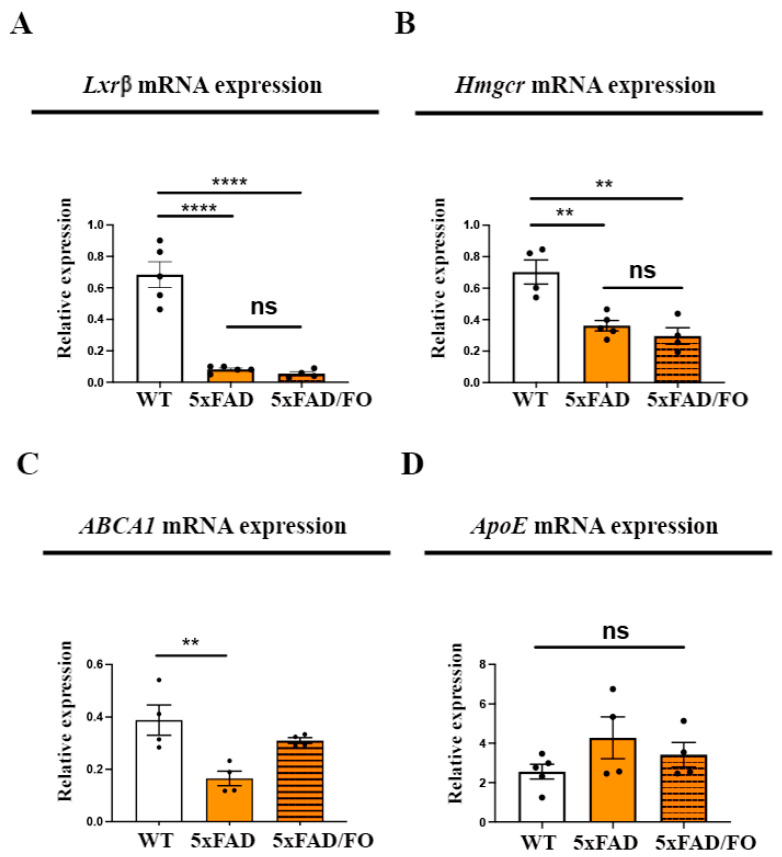
High-dose FO supplementation did not alter the expression of the genes involved in regulating cholesterol synthesis and transport in 5xFAD retinas. Lxrβ (**A**), Hmgcr (**B**), ABCA1 (**C**), and ApoE (**D**) mRNA expression in the control, 5xFAD, and 5xFAD/FO retinas. Data were analyzed using one-way ANOVA (with Tukey’s post hoc). The results are presented as mean ± SEM value. ** *p* < 0.01, **** *p* < 0.0001.

The endothelial cells’ lipid content is essential in regulating transcytosis and barrier permeability [13] and influencing the expression and distribution of Aqp4 [48]. Additionally, changes in the expression of genes involved in cholesterol synthesis have been detected in Mfsd2a^−/−^ retinas [29,46], implying a potential feedback regulation. We examined the changes in the expression of the key genes regulating cholesterol synthesis in the 5xFAD and WT retinas: liver X receptor beta (Lxrβ) and endoplasmic reticulum-bound 3-hydroxy-3-methylglutaryl-coenzyme A reductase (Hmgcr). *Lxrβ* was shown to integrate cholesterol input and output pathways [49], while *Hmgcr* is considered the rate-limiting enzyme in cholesterol synthesis [50]. A qRT-PCR analysis showed that *Lxrβ* and *Hmgcr* expression levels were significantly lower in the 5xFAD retinas in comparison to the control mice (8.3-fold and 1.9-fold decrease, respectively, Figure 2A,B). Therefore, it is unsurprising that the FO supplementation did not further lower the expression levels of these genes in the 5xFAD retinas (Figure 2A,B). 

Next, we aimed to determine whether FO supplementation altered the expression of genes involved in cholesterol turnover. Efficient recycling of cholesterol in the cells is mediated via a mechanism that is apolipoprotein-dependent, and ApoE is the most prominent apolipoprotein [51]. The ATP-binding cassette transporter A1 (ABCA1) facilitates this process and is responsible for the efflux of cholesterol and phospholipids [51]. The qRT-PCR analyses showed that the *ABCA* expression levels were decreased (2.3-fold decrease, Figure 2C), but the levels of *ApoE* were unaltered in 5xFAD retinas (Figure 2D). The supplementation with FO did not significantly alter *ABCA* (Figure 2C) or *ApoE* (Figure 2D) expression in the 5xFAD retinas. 

### 2.3. The High-Dose FO Supplementation Increased Mfsd2a Blood Vessels Coverage in 4M 5xFAD Retinas

**Figure 3:**
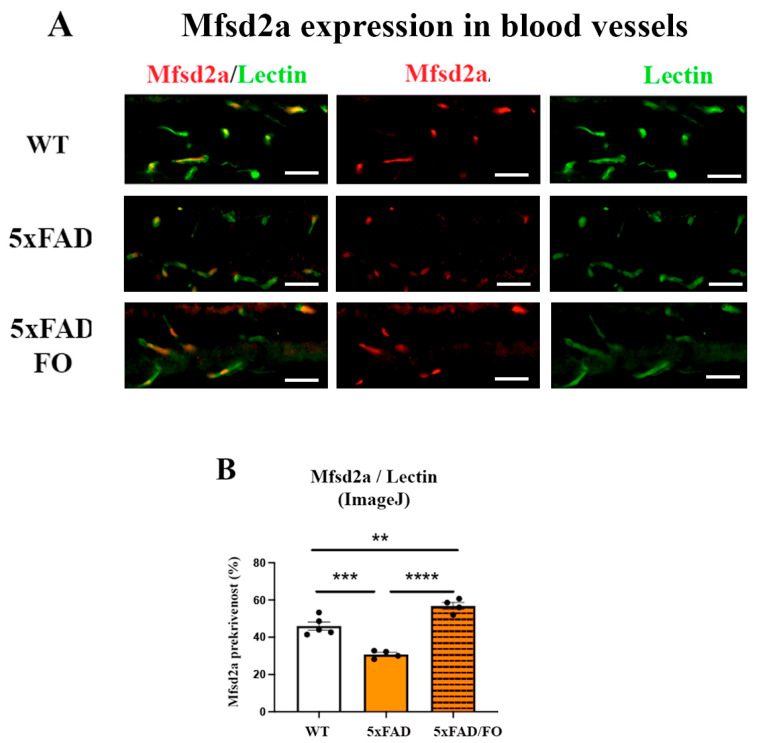
The high-dose FO supplementation increased the Mfsd2a blood vessel coverage in 4M 5xFAD retinas. (**A**) The Mfsd2a coverage of the retinal blood vessel (Mfsd2a—red; Lectin—green; DAPI—blue). (**B**) The quantification of the Mfsd2a coverage of the retinal blood vessel (graph). Scale bar—50 μm. Data were analyzed using one-way ANOVA (with Tukey’s post hoc). The results are presented as mean ± SEM value. ** *p* < 0.01, *** *p* < 0.001, **** *p* < 0.0001.

The expression of the retinal Mfsd2a is mainly observed in endothelial cells [52]. The analyses of Mfsd2a expression (anti-Mfsd2a antibody, labeled in red) in retinal blood vessels (anti-Lectin, labeled in green) were conducted in all experimental groups (Figure 3A). A decreased Mfsd2a blood vessel coverage in the 5xFAD retinas was revealed using ImageJ (Version 2.14.0/1.54f) analyses (1.31-fold decrease, Figure 3B). The assessment of the Mfsd2a coverage of retinal blood vessels was presented as the ratio of Mfsd2a/Lectin staining (ImageJ, Figure 3B). In contrast, the FO supplementation significantly increased Mfsd2a coverage in 5xFAD retinas (1.8-fold increase compared to 5xFAD, 1.23-fold increase compared to the control, Figure 3B).

### 2.4. The High-Dose FO Supplementation Reduced the Levels of Aqp4 Expression in 4M 5xFAD Retinas

**Figure 4:**
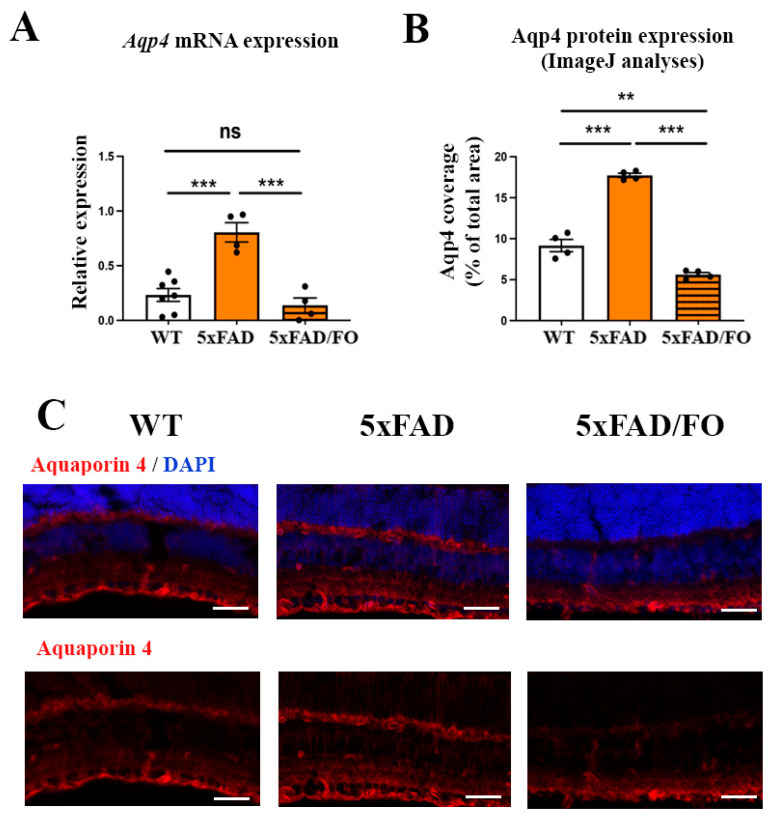
The high-dose FO supplementation reduced the levels of Aqp4 expression in 4M 5xFAD retinas. (**A**) Aqp4 mRNA expression. (**B**) The representative images of Aqp4 expression in the 5xFAD and 5xFAD/FO retinas. (Aqp4—labeled in red; DAPI—labeled in blue). (**C**) The quantification of the Aqp4 protein expression (ImageJ). Scale bar—50 μm. Data were analyzed using one-way ANOVA (with Tukey’s post hoc). The results are presented as mean ± SEM value. ** *p* < 0.01, *** *p* < 0.001.

The *Aqp4* expression levels were shown to increase in the brains [22] and retinas [17] of the 5xFAD mice with age and the progression of the disease, indicating the impairment of the glymphatic system. Indeed, Aqp4 mRNA expression levels were elevated in the 5xFAD retinas (3.52-fold increase, Figure 4A). However, the FO supplementation was shown to suppress Aqp4 expression in the model of traumatic brain injury [31]. We, therefore, analyzed Aqp4 mRNA expression in the FO-supplemented 5xFAD retinas. Aqp4 mRNA levels were decreased in the FO-supplemented 5xFAD retinas when compared to the untreated 5xFAD mice (5.8-fold decrease, Figure 4A). Similar changes in the expression levels of the Aqp4 protein were observed using immunohistochemical staining with the Aqp4 antibody (depicted in red, Figure 4C). The ImageJ analyses of the immunohistochemical staining of retinal sections revealed an increase in Aqp4 protein expression in the 5xFAD retinas (1.95-fold increase in comparison to controls, Figure 4B), which was reduced following the FO supplementation (1.64-fold decrease compared to the controls, 3.2-fold decrease compared to 5xFAD, Figure 4B).

### 2.5. The High-Dose FO Supplementation Did Not Alter the Perivascular Aqp4 Expression in FO Supplemented 4M 5xFAD Retinas

**Figure 5:**
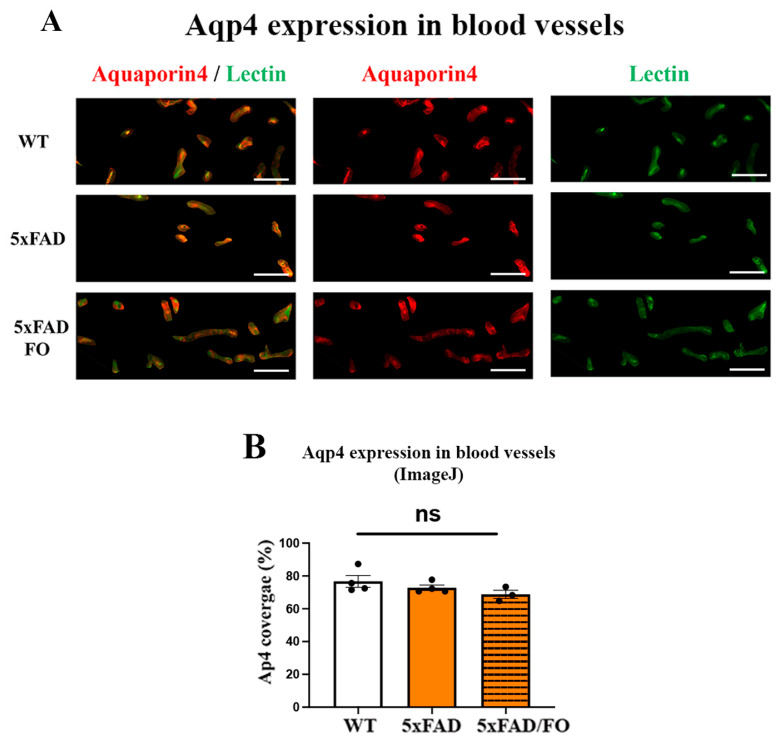
Perivascular Aqp4 expression is not altered in FO-supplemented 5xFAD retinas. (**A**) The Aqp4 perivascular expression in the 4M 5xFAD and 5xFAD/FO retinas (anti-Aqp4 antibody—labeled in red; anti-Lectin antibody—labeled in green; DAPI—labeled in blue). (**B**) The assessment of the perivascular Aqp4 localization (graph). Scale bar—50 μm. Data were analyzed using one-way ANOVA (with Tukey’s post hoc). The results are presented as mean ± SEM value; ns: nonspecific.

Impaired glymphatic clearance has been observed in the AD and senescence animal models [53], affecting Aqp4 expression around blood vessels and disrupting the polarization of perivascular Aqp4. Moreover, a reduction in perivascular Aqp4 expression is significantly linked to an increased Aβ burden [23]. We analyzed the perivascular Aqp4-staining (Aqp4 staining in red and lectin staining in green) in the control, 5xFAD, and 5xFAD–FO retinas and did not observe any changes (Figure 5B). The assessment of the Aqp4 perivascular localization was presented as the ratio of Aqp4/Lectin staining.

### 2.6. The High-Dose FO-Supplementation Decreased Amyloid β Accumulation in Retinal Blood Vessels in 4M 5xFAD Mice

**Figure 6:**
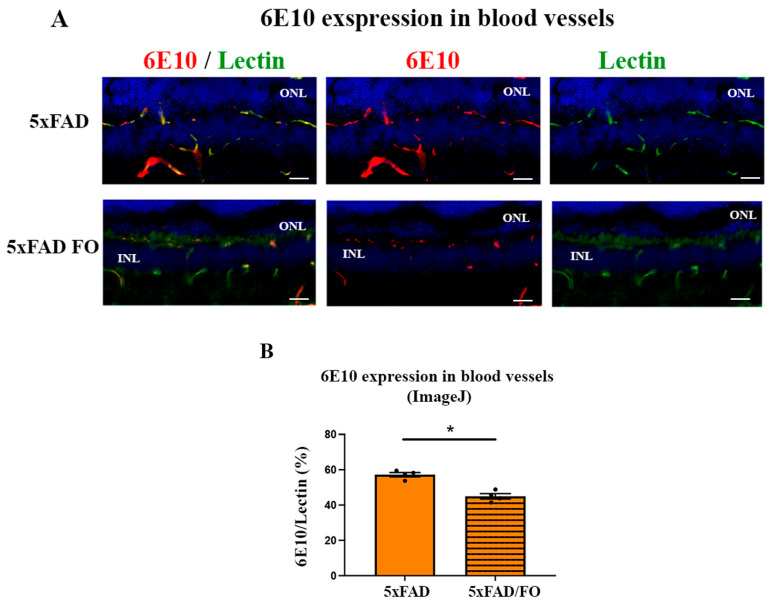
The high-dose FO-supplementation decreased Ab accumulation in retinal blood vessels in the 4M 5xFAD mice. (**A**) Aβ expression in the retinal blood vessels (anti-Aβ antibody, 6E10—labeled in red; anti-lectin antibody—labeled in green; DAPI—labeled in blue). (**B**) The quantitative analyses of Aβ accumulation in retinal blood vessels (graph). Scale bar—50 μm. Data were analyzed using the nonparametric Mann–Whitney U test. The results are presented as mean ± SEM value. * *p* < 0.05. ONL—outer nuclear layer, INL—inner nuclear layer.

Aβ accumulation was observed in the blood vessels in the brain [38] and retina [17] from the 5xFAD mice. Therefore, we attempted to quantify the effect of the FO supplementation on the levels of Aβ accumulation in the retinal blood vessels using immunohistochemistry (Figure 6A). Double labeling with 6E10 (anti-Aβ antibody) and lectin showed a strong colocalization of Aβ (red) in retinal blood vessels (green) from the 4M 5xFAD retinas (Figure 6A). The changes in the expression levels of Aβ in retinal blood vessels were assessed as the ratio of Aqp4/Lectin staining. Our analyses showed that FO supplementation significantly decreased Ab expression levels in the retinal blood vessels (21% decrease, Figure 6B).

## 3. Discussion

In AD, a compromised BBB function is considered a primary factor in cerebral amyloidosis, as it plays a crucial role in clearing excessive cerebral Aβ [54,55]. The accumulation of amyloid in blood vessels is attributed to inadequate clearance of Aβ and is linked to impaired transcytosis and dysfunction in the glymphatic system. Recent findings revealed that supplementation with n-3 LC-PUFAs, such as EPA and DHA, reduced Aβ levels in the retina of 5xFAD mice [42]. However, the exact mechanisms involved in this process are not entirely understood. In the present study, the high-dose short-term (3 weeks long) FO supplementation of 4M5xFAD mice significantly increased the expression levels of the mRNA of the primary regulator of transcytosis, *Mfsd2a.* At the same time, we observed an increase in the Mfsd2a retinal blood vessel coverage. Simultaneously, the observed increased expression of Aqp4 in 5xFAD retinas was downregulated following the FO supplementation. Nevertheless, the expression levels of the genes regulating cholesterol metabolism in the retina were not significantly altered. Finally, after the FO supplementation, we observed a 21% reduction in Aβ accumulation in the 5xFAD retinal blood vessels.

The commonly recommended dosage of omega-3 fatty acid supplementation is 450–500 milligrams/day [56,57]. However, current observations revealed that low DHA bioavailability can result from the lower dosage of supplementation, thus significantly diminishing the beneficial effects of FO [32]. As a result, doses up to 10 g/day are suggested [32]. This is supported by the EFSA Panel on Dietetic Products, Nutrition, and Allergies [57], which stated that high-dose omega-3 supplementation (up to 5 g/day, up to 16 weeks) could be safe. Consequently, higher doses of omega-3 fatty acids are already advocated as treatments in patients with mild traumatic brain injury (mTBI) [58], as the therapy during the early period of major depressive disorder (MDD) [59], as a potential therapeutic for abnormal retinal neovascularization [60,61], and as anti-amyloidogenic agent in AD animal models [35,36]. Our previous study revealed that such higher FO dosage increased the expression of Mfsd2a in the retinal blood vessels in the WT mice, suggesting its protective effect on the function of BRB [34].

Reduced clearance of Aβ from the brain parenchyma, accompanied with impaired amyloid transport and degradation, are characteristics of CAA [62,63,64]. The retinal blood vessels Aβ accretion observed in 5xFAD mice is similar to the previously reported vascular amyloidosis in other AD transgenic mice (APPSWE/PS1ΔE9 and Tg2576 mouse) [9,65], and in postmortem analyses of the MCI and AD human retinas [10,11]. However, although changes in vascular function and structure in the 5xFAD retinas were reported by us [17] and others [66,67], the opposite findings were also described [68].

We have previously reported the changes in the expression levels of Mfsd2a and Aqp4 in 5xFAD mice that were concomitant with aging and the progression of AD pathology [17]. Mfsd2a is essential for controlling transcytosis in CNS endothelial cells of the BBB, as demonstrated by increased transcytotic vesicles observed in endothelial cells of Mfsd2a-deficient mice, leading to BBB leakage [16] before any morphological changes or changes in the expression of tight junction proteins [13]. Such a substantial decrease in the Mfsd2a expression in 5XFAD retinas, indicative of the compromised BRB, suggests that the deregulation of Mfsd2a and impaired transcytosis may be the reason for the reduced Aβ clearance from the blood vessels. Mfsd2a downregulation was also reported in diabetic retinopathy and was associated with the upregulation of the Srebp signaling pathway [46], that was previously observed in the eyes of Mfsd2a knockout mice [69]. We observed the simultaneous upregulation of srebp-1c, in parallel with the downregulation of Mfsd2a in 4M 5xFAD retinas. Following the FO supplementation, the expression levels of *Mfsd2a* in the 5xFAD retinas were significantly increased, as was previously observed in the retinas of the FO-supplemented WT mice [34]. At the same time, the FO supplementation decreased the expression levels of *Srebp1* concomitantly with the observed increase in Mfsd2a expression, suggesting one possible mode of Mfsd2a expression regulation in the 5xFAD retinas. 

Endothelial cells’ lipid composition plays a role in regulating transcytosis and barrier permeability [13], as well as influencing the expression and distribution of Aqp4 [48]. Additionally, the changes in the expression levels of the genes regulating cholesterol synthesis in Mfsd2a^−/−^ retinas [15,46] suggest a likely co-regulation. The proposed mechanism indicates that the increased levels of DHA, transported by Mfsd2a [28], can displace cholesterol and Cav-1 from the membrane and inhibit the formation of caveolae consequently inhibiting transcytosis [70]. Accordingly, Lxr agonist T0901317 was able to induce Mfsd2a upregulation [71]. In addition, chromatin immunoprecipitation sequencing (ChIPseq) together with gene array studies identified Lxrb binding sites within intron 3 of the mouse Mfsd2a gene [72]. The decreased *Lxrb* expression in the 5xFAD retinas concomitant with Mfsd2a downregulation observed in this study implicates another possible mechanism of Mfsd2a expression regulation. The expression *Hmgcr*, the critical factor in cholesterol synthesis, was also downregulated in accordance with the observed attenuation of cholesterol metabolism in the CNS, characteristic for 5xFAD mice [73]. Nevertheless, understanding how dysregulated synthesis of cholesterol is affecting Mfsd2a expression levels and, consequently, the transcytosis in the retina warrants additional research. As the levels of *Lxrb* and *Hmgcr* expression were already significantly decreased in the 5xFAD retinas compared to WTs, FO supplementation could not exert its lowering effect.

It has been proposed that Mfsd2a manipulations may influence transcytotic mechanisms in CNS endothelial cells for therapeutic applications [74]. Recent studies have revealed that storax-induced attenuation of BBB impairment, which helps to halt the progression of cerebral ischemia [75], occurs through the upregulation of Mfsd2a and concurrent inhibition of Cav-1 in the endothelial cells. In addition, LPC-DHA, which is transported via Mfsd2a, significantly improved 5xFAD retinal function [42]. Treatment with FO also notably increased Mfsd2a expression together with Mfsd2a blood vessel coverage in the WT retinas [34]. Furthermore, overexpression of Mfsd2a in the retina of mouse models of retinopathy reduced neovascularization and vascular leakage, with a synergistic effect observed when co-treated with DHA [46].

Aqp4 is another key regulator of Aβ clearance, present in glia encasing the blood vessels [76,77]. Aqp4 has an important role in regulating the exchange of cerebrospinal fluid (CSF) and interstitial fluid around the blood vessels. The aging [22] and the progression of AD pathology [17,22] was shown to increase its levels in the retina. The FO supplementation restored the increased Aqp4 levels in the treated 5xFAD retinas to the control levels. Importantly, it was shown that the active role of the glymphatic system is necessary in promoting the beneficial outcomes of n-3 PUFA supplementation on Aβ elimination [30]. The lack of any changes in the perivascular Aqp4 localization was expected, considering that the study was conducted in the presymptomatic phase of the disease and that the changes in perivascular localization are associated with aging [53] and the progression of AD pathology [17,22,76]. 

The exact mechanism by which Aqp4 influences Aβ accumulation in blood vessels via glymphatic system remains unclear. Deletion of Aqp4 has been shown to reduce Aβ clearance, leading to increased accumulation of Aβ peptides in plaques and blood vessels in AD transgenic mice [78]. However, the use of Aqp4 inhibitors, such as TGN-020, markedly increased Aβ accretion [79]. Furthermore, fish oil treatment has been demonstrated to preserve Aqp4 polarization while significantly improving Aβ clearance from the brain [30]. These findings suggesting the potential for targeted manipulation of the glymphatic system. 

Although the molecular changes observed in this study are linked to the effects of FO supplementation, detailed analyses of fatty acid content changes in the retina are missing. Given that DHA levels are reduced in 5xFAD retinas [42], the absence of these data represents a limitation of the study. Additionally, comparative analyses of molecular changes in different regions of the retinal blood vessel network in FO-supplemented 5xFAD mice have yet to be conducted. It is possible that FO components may have varying access to different parts of the retina.

It was shown that the supplementation with DHA and EPA in the triacylglycerol form was able to induce a 17% reduction in Aβ accumulation in the retinal tissue [42]. This study explores the changes in the Aβ accumulation following the FO supplementation, specifically in the retinal blood vessels, implicating the impaired transcytosis and glymphatic system function as culprits. The increase in Mfsd2a expression and decrease in Aqp4 expression following the FO supplementation were concomitant with the 22% reduction in the Aβ accumulation in retinal blood vessels in 5xFAD mice. These findings implicate Mfsd2a and Aqp4 as putative targets for new and adjuvant therapies. This is especially significant because Aβ accumulation in retinal blood vessels is shared by several age-related neurodegenerative disorders—AD, glaucoma, and age-related macular degeneration (AMD)—all of which currently lack a cure [80]. Therefore, developing new therapies that target impaired transcytosis and glymphatic system is urgently needed. This is particularly important considering that Aβ deposition can start early, in the presymptomatic phase of the disease, and can last for decades before the manifestation of AD hallmarks. Thus, the concurrent increase in Mfsd2a expression and reduction in Aqp4 expression following the high-dose FO supplementation resulting in the decreased blood vessels Aβ accretion in the retina indicates the strong potential of the FO as an adjuvant therapy in the presymptomatic phase of AD or other amyloid-based diseases.

## 4. Materials and Methods

### 4.1. Animals

All animal procedures in this study adhered to the Directive (2010/63/EU) on the safeguarding of animals used for scientific reasons. The procedures were permitted by the Ethical Committee for the Use of Laboratory Animals (resolution No. 01-06/13, Institute for Biological Research, University of Belgrade) and complied with the EEC Directive (86/609/EEC) on the protection of animals. The control and 5xFAD mice were housed in typical settings (12 h light/dark cycle, 23 ± 2 °C, relative humidity: 60–70%) and were routinely monitored for health. Pelleted commercial rodent chow was provided ad libitum (AL).

The 5xFAD mice (Jackson Laboratory, Cat. No: 3484-JAX and 100012-JAX, Bar Harbor, ME, USA) were maintained on a B6/SJL genetic background (5xFAD transgenic male mice × B6SJLF1/J female mice). Females (F1 generation) were used for the analyses. The 5xFAD animals overexpress mutated human APP 695, with the following mutations: Swedish mutation (K670N, M671L), Florida mutation (I716V), London mutation (V717I), and human presenilin 1 with 2 FAD mutations (M146L and L286V) [34].

### 4.2. Treatment

Female B6/SJL (WT) and 5xFAD mice (three-month-old) that were fed on commercial rodent chow (Table 1) were used for treatment with the fish oil. The WT and 5xFAD mice were divided into two groups—the FO-treated group (*n* = 11), which received FO (DietPharm, FidaFarm, Zagreb, Croatia), and the control group (*n* = 11), which was given an equal volume of water. The treated group was administered 100 µL of fish oil daily via oral gavage for 21 days (fatty acid composition in Table 2). The dose in this study is considered a high-dose DHA or EPA supplementation. Briefly, animal equivalent dose (AED) (mg/kg) is calculated as human dose (50 mg/kg, which amounts to 3000 mg/day of DHA) multiplied by the correction factor for mice (Km): AED (mg/kg) = 50 mg/kg × 12.3 = 615 mg/kg. Therefore, 545.5 mg/kg DHA and 818.2 mg/kg of EPA were given to the animals daily through the supplementation with 12 mg DHA and 18 mg EPA per 100 μL.

### 4.3. Tissue Collection

Four-month-old animals were anesthetized (Ketamidor, 100 mg/kg, Richter Pharma, Wells, Austria and Xulased, 16 mg/kg, Bioveta, a.s. administered intraperitoneally), perfused with 50 mL 0.1 M phosphate buffer (PBS, 30 min), and then decapitated. After enucleation of the eyes and removal of the cornea, lens, and vitreous body, the retina was isolated and stored at −80 °C for further analyses: *n* = 7/per group for qPCR. Four eyes from each group were collected for immunohistochemistry.

### 4.4. Real-Time Quantitative Polymerase Chain Reaction (qRT-PCR)

#### 4.4.1. RNA Isolation and Reverse Transcription

The total RNA was isolated from the retinas obtained from the control, 5xFAD, and 5xFAD FO 4-month-old animals (*n* = 7/group, TRIZOL isolation system, Invitrogen Life Technologies, Carlsbad, CA, USA). The concentration of pelleted RNA, dissolved in 20 μL was assessed by spectrophotometry and its integrity was confirmed using 1% agarose gel electrophoresis. Six micrograms of total RNA were treated with RNase-free DNase I (Thermo Fisher Scientific, Waltham, MA, USA) and reverse transcribed (High-Capacity cDNA Archive Kit, Applied Biosystems, Fister City, CA, USA). cDNA was stored at −20 °C for analyses.

#### 4.4.2. Quantitative RT-PCR (qRT-PCR)

In total, 20 ng of the resulting cDNA was used for the PCR analysis in a final volume of 10 μL using RT2 SYBR Green qPCR Mastermix (Applied Biosystems, Fister City, CA, USA). RT-PCR amplifications (ABI 7500 thermal cycler, Applied Biosystems, Fister City, CA, USA) in the default cycling mode (50 °C for 30 min, 95 °C for 15 min, followed by 40 cycles of 94 °C for 60 s, 57 °C for 60 s, 72 °C for 60 s, 70 °C for 10 min). The results obtained by qRT-PCR were analyzed in an RQ Study add-on software for the 7000 v 1.1 SDS instrument (confidence level of 95%, *p* < 0.05). Quantification of mRNA levels was performed using the 2-DDCt method [81] and expressed relative to the control. Sequences of the used primers (by Vivogen, Belgrade, Serbia) are given in Table 3.

### 4.5. Immunohistochemistry

The enucleated eyes were fixed in 4% paraformaldehyde at 4 °C (O/N), cryoprotected in 7.5% gelatin and 15% sucrose. For the analyses, 18 µm cryo-sections were prepared. Sections were incubated at 37 °C for 30 min in 1xPBS, permeabilized (0.5% Triton/1xPBS, 1xPBST, 15 min), and then blocked (1% bovine serum albumin, BSA, in 1xPBST, 1 h) at RT. Overnight incubation with primary antibodies: mouse monoclonal 6E10 (cat#803003, Biolegend, San Diego, CA, USA), rabbit polyclonal Aqp4 (1:500, AB3594, EMD Millipore, Burlington, MA, USA), and rabbit-polyclonal Mfsd2a primary antibody (1:10,000, 10,539, Abcam, Cambridge, UK) were conducted at 4 °C. Furthermore, 488-conjugated Lycopersicon esculentum lectin (488 DL1174, Vector, Newark, CA, USA) was used to label blood vessels. Following the washing (1xPBST) and incubation with 568-conjugated anti-rabbit and anti-mouse secondary antibodies (Invitrogen, Carlsbad, CA, USA, 1:500, 2 h RT) slides were mounted with DAPI mounting medium (Merck, Darmstadt, Germany) and analyzed using fluorescent microscopy. Micrographs were captured on an Axio Observer Microscope (Z1 AxioVision 4.6 software system, Carl Zeiss, Oberkochen, Baden-Wurttemberg, Germany) at 20×. The images shown are representative of observations from 3 independent stainings per group (*n* = 4). In all images, the apical side is oriented upwards.

### 4.6. Quantification of Perivascular Aqp4 Expression

Quantification was performed from representative images taken on a Zeiss microscope at 20× magnification with conditions kept identical for all groups. Aqp4- and the lectin-positive area were measured using threshold processing (ImageJ software, 2.14.0/1.54f, NIH, USA), and the perivascular Aqp4 expression was expressed as the ratio between the area occupied by Aqp4 and the area occupied by lectin (the result is expressed as the % of occupied area). In each animal (*n* = 4 per group), five fields from 2 to 4 retinal sections were analyzed.

### 4.7. Statistical Analysis

Data were analyzed using Prism program (GraphPad Prism, Software, v.6, La Jolla, CA, USA). A one-way ANOVA (with Tukey’s post hoc test) was used to establish statistically significant differences between three experimental groups. The nonparametric Mann–Whitney U test was used to compare two experimental groups, since that data did not meet the normal distribution criteria. The test was considered significant when *p* < 0.05.

## Figures and Tables

**Table 1:** Commercial diet content (pellets).

Component	%
Protein	17.2
Carbohydrate	60.9
Fat	3.7
PUFA/SFA	1.3
n-3/n-6 PUFA	0.05
fiber	5.6
ash	7.6
Vitamins and minerals (in adequate amounts)	

PUFA, polyunsaturated fatty acids; SFA, saturated fatty acids; n-3, omega-3; n-6, omega-6.

**Table 2:** Fatty acid composition of fish oil (% *w*/*w* of total fatty acids).

SFA	16:0n	palmitic	22.90
	18:0n	stearic	2.23
MUFA	16:1n-7	palmitoleic	11.90
	18:1n-7	vaccenic	4.54
n-6	18:2n-6	linoleic	1.67
	20:3n-6	dihomo-gama-linolenic	0.29
	20:4n-6	arachidonic	1.62
	22:4n-6	adrenic	1.78
n-3	20:5n-3	EPA	25.51
	22:5n-3	DPA	1.82
	22:6n-3	DHA	15.49

SFA, saturated fatty acids; MUFA, mono-unsaturated fatty acids; n-6, omega-6 (PUFA); n-3, omega-3; EPA, eicosapentaenoic acid; DPA, docosapentaenoic acid; DHA, docosahexaenoic acid.

**Table 3:** Primer sequences for expression studies.

Gene	Orientation	Sequence
*HMGCR*	F (5′-3′)	TTG GTC CTT GTT CAC GCT CAT
	R (3′-5′)	TTC GCC AGA CCC AAG GAA AC
*SREBP1-C*	F (5′-3′)	ACG GAG CCA TGG ATT GCA
	R (3′-5′)	AAG TCA CTG TCT TGG TTG TTGATGA
*LXRBETA*	F (5′-3′)	AGC GTC CAT TCA GAG CAA GTG
	R (3′-5′)	CAC TCG TGG ACA TCC CAG ATC T
*ABCA*	F (5′-3′)	AGG CCG CAC CAT TAT TTT GTC
	R (3′-5′)	GGC AAT TCT GTC CCC AAG GAT
*APOE*	F (5′-3′)	GGC CCA GGA GGA GAA TCA ATGA G
	R (3′-5′)	CCT GGC TGG ATA TGG ATG TTG
*MFSD2A*	F (5′-3′)	AGA AGC AGC AAC TGT CCA TTT
	R (3′-5′)	CTC GGC CCA CAA AAA GGA TAA T
*HPRT*	F (5′-3′)	CTC ATG GAC TGA TTA TGG ACA GGA C
	R (3′-5′)	GCA GGT CAG CAA AGA ACT TAT AGC C
*AQP4*	F (5′-3′) R (3′-5′)	AGC AAT TGG ATT TTC CGT TG TGA GCT CCA CAT CAG GAC AG

F—forward primer, R—reverse primer.

## Data Availability

The original contributions presented in the study are included in the article, further inquiries can be directed to the corresponding author.
